# N-acetylcysteine Amide Ameliorates Blast-Induced Changes in Blood-Brain Barrier Integrity in Rats

**DOI:** 10.3389/fneur.2019.00650

**Published:** 2019-06-26

**Authors:** Usmah Kawoos, Rania Abutarboush, Sydney Zarriello, Aasheen Qadri, Stephen T. Ahlers, Richard M. McCarron, Mikulas Chavko

**Affiliations:** ^1^The Henry M. Jackson Foundation for the Advancement of Military Medicine, Inc., Bethesda, MD, United States; ^2^Department of Neurotrauma, Naval Medical Research Center, Silver Spring, MD, United States; ^3^Morsani College of Medicine, University of South Florida, Tampa, FL, United States; ^4^Department of Biology, University of Maryland, College Park, MD, United States; ^5^Department of Surgery, Uniformed Services University of the Health Sciences, Bethesda, MD, United States

**Keywords:** repetitive blast, blood-brain barrier, N-acetylcysteine amide, intravital microscopy, fluorescent imaging

## Abstract

Traumatic brain injury resulting from exposure to blast overpressure (BOP) is associated with neuropathology including impairment of the blood-brain barrier (BBB). This study examined the effects of repeated exposure to primary BOP and post-blast treatment with an antioxidant, N-acetylcysteine amide (NACA) on the integrity of BBB. Anesthetized rats were exposed to three 110 kPa BOPs separated by 0.5 h. BBB integrity was examined *in vivo* via a cranial window allowing imaging of pial microcirculation by intravital microscopy. Tetramethylrhodamine isothiocyanate Dextran (TRITC-Dextran, mw = 40 kDa or 150 kDa) was injected intravenously 2.5 h after the first BOP exposure and the leakage of TRITC-Dextran from pial microvessels into the brain parenchyma was assessed. The animals were randomized into 6 groups (*n* = 5/group): four groups received 40 kDa TRITC-Dextran (BOP-40, sham-40, BOP-40 NACA, and sham-40 NACA), and two groups received 150 kDa TRITC-Dextran (BOP-150 and sham-150). NACA treated groups were administered NACA 2 h after the first BOP exposure. The rate of TRITC-Dextran leakage was significantly higher in BOP-40 than in sham-40 group. NACA treatment significantly reduced TRITC-Dextran leakage in BOP-40 NACA group and sham-40 NACA group presented the least amount of leakage. The rate of leakage in BOP-150 and sham-150 groups was comparable to sham-40 NACA and thus these groups were not assessed for the effects of NACA. Collectively, these data suggest that BBB integrity is compromised following BOP exposure and that NACA treatment at a single dose may significantly protect against blast-induced BBB breakdown.

## Introduction

Blast-induced traumatic brain injury (bTBI) has been of particular concern with the rise of improvised explosive devices in warzones (1, 2). Recent research has shown that one mechanism of injury related to bTBI is the compromise of blood-brain barrier (BBB) integrity following blast exposure (3–5). BBB is an anatomical structure formed of endothelial cells connected by tight junction (TJ) proteins, pericytes, basement membrane, and astrocytic end feet. It plays a key role in maintaining brain solute homeostasis, and providing protection against potentially toxic substances in the circulation from reaching the central nervous system. Endothelial TJs prevent the diffusion of metabolites and polar blood-borne substances from reaching brain tissue (6). Oxidative stress is a contributing factor in the increase in permeability of the BBB via the alteration of TJ structure (7). Though the mechanisms are unclear, oxidative stress characterized by a decrease in glutathione (GSH) levels in brain tissue was shown in rats after bTBI exposure (8). BBB structural integrity is maintained by a variety of protein molecules such as occludin, junction associated protein-1 (JAM-1), and claudin-5, which all co-localize with the primary TJ peripheral protein, zona occludens (ZO-1) (9, 10). The changes in occludin assembly localization and structure at TJs are strongly associated with increased BBB permeability due to oxidative stress (7, 11). After exposure to bTBI, rat brain endothelial cells demonstrated decreased trans-endothelial electrical resistance and increased water flux through TJs, indicating increased BBB permeability (3). The extent of permeability changes in BBB due to bTBI depends on intensity and repetition of blasts, separation between them, and the time after blast(s). Studies have shown extravasation of small- and large-sized permeability markers (Evans Blue, sodium fluorescein, IgG) 24 h after exposure to single or repetitive blasts (5, 12). Other studies have also presented the extent of BBB opening to be dependent on time after blast (13). Dysfunction of the BBB and subsequent leakage of blood-borne proteins has been shown to lead to cerebral edema (14). Vasogenic edema is related to increased intracranial pressure (ICP), which has been associated with bTBI in the hours and days following injury (5, 15). Post-blast treatment with an antioxidant was shown to alleviate blast-induced increase in ICP (16). Prevention of the disruption of BBB by minimization of oxidative stress is a potential therapeutic target for bTBI.

*N*-acetylcysteine (NAC), a cysteine precursor molecule, enhances the production of endogenous antioxidant, GSH which plays an important role in maintaining the cellular redox state (17, 18). Recent studies have reported prophylactic action of NAC in improving the outcome after TBI (19–23). However, the bioavalibility of NAC is inadequate due to its limited penetration through the BBB (24). The carboxyl group in NAC is replaced with an amide to form *N*-acetylcysteine amide (NACA), conferring increased lipophilicity and permeability through BBB compared to NAC (24). This and our findings that NACA ameliorates post-blast ICP increase has warranted the potential use of NACA, a thiol antioxidant (25, 26), as a therapeutic agent after blast exposure.

This study presents an *in vivo* assessment of the extent of blast-induced damage to BBB permeability in acute phase after repeated blasts by utilizing small- and large-sized permeability markers and the efficacy of NACA in ameliorating the damage to BBB.

## Materials and Methods

The study protocol was reviewed and approved by the Walter Reed Army Institute of Research/Naval Medical Research Center Institutional Animal Care and Use Committee in compliance with all applicable federal regulations governing the protection of animals in research. The experiments reported herein were conducted in compliance with the Animal Welfare Act and per the principles set forth in the “Guide for Care and Use of Laboratory Animals,” Institute of Laboratory Animals Resources, National Research Council, National Academy Press, 2011.

### Experimental Groups

**Figure 1:**
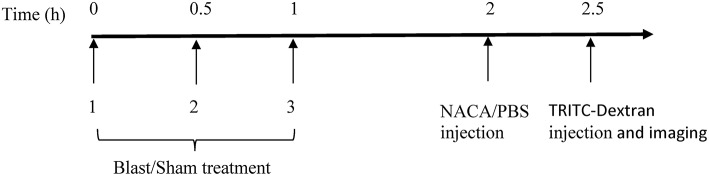
Timeline of the experimental procedures.

Adult male Sprague-Dawley rats (300–350 g; 8–9 weeks; Taconic Farms, NY, USA) were randomly assigned to one of the 6 groups (*n* = 5/group): four groups received 40 kDa tetramethylrhodamine isothiocyanate (TRITC) Dextran (BOP-40, sham-40, BOP-40 NACA, and sham-40 NACA), and two groups received 150 kDa TRITC-Dextran (BOP-150 and sham-150). To visualize any compromise in BBB, the animals were administered a 25 mg/kg intravenous injection of TRITC-Dextran (Sigma-Aldrich, MW = 40 kDa or 150 kDa) 2.5 h after the first BOP exposure. Animals treated with NACA (David Pharmaceuticals, New York, NY, USA) were administered an intraperitoneal (IP) injection of the antioxidant [500 mg/kg/1.5 ml phosphate buffer solution (PBS)] 2 h after the first BOP exposure. A high dose of NACA was selected based on our previous study where this dose resulted in attenuation of blast-induced increase in ICP (16). Animals that did not receive NACA were treated with PBS (1.5 ml/kg). The timeline of procedures is presented in Figure 1.

### Blast Exposure

All animals assigned to BOP groups were anesthetized with isoflurane (5%) prior to BOP exposure in frontal orientation, i.e., head facing and body parallel to the blast wave, in a cylindrical shock tube (27). The shock tube consists of compression and expansion chambers separated by a polyethylene Mylar™ membrane (DuPont Teijin Films™, Wilmington, DE, USA). Anesthetized animals were secured in an animal retainer designed to stabilize the animal and minimize any movement during blast exposure to circumvent infliction of secondary injury due to movement. Blast animals were exposed to three BOPs separated by 0.5 h. The mean (standard deviation) peak pressure, duration of positive phase, and impulse during the positive phase of BOP were 110 (3) kPa, 7.1 (0.06) ms, and 0.32 (0.002) kPa.s, respectively. Sham animals were anesthetized with isoflurane but were not subjected to BOP exposure.

### Intravital Microscopy and Imaging of Pial Microcirculation

Rats were anesthetized with IP administration of ketamine (70–85 mg/kg) and xylazine (8–12 mg/kg), which were re-administered as needed. Pial microcirculation was exposed via a rectangular craniotomy (~2 × 4 mm) in the right parietal bone followed by resection of dura mater as previously described (28). Brain surface electrolyte balance was maintained with topical administration of artificial cerebrospinal fluid (Harvard Apparatus, Cambridge, MA, USA). The microvessels were visualized with a stereomicroscope (SZ16, Olympus, Japan) equipped with a DP-73 digital camera. The extravasation of TRITC-Dextran from pial microvessels into brain parenchyma was imaged over 5 min. All animals were euthanized with 1 mL/kg IP Euthasol (Visbac®, Fort Worth, TX) at the end of the observation period.

### Data and Statistical Analysis

BBB leakage was identified by an increase in the fluorescence intensity in close proximity to pial vessels. Qualitatively, the extravasation of TRITC-Dextran from pial vessels was determined by the change in intensity of fluorescence outside of the pial vessels over a period of 5 min. Specifically, leakage was visualized from the difference of images acquired 5 min and 5–10 s (baseline or t_0_) after the administration of the probe. The quantitative analysis of impaired BBB permeability was performed by estimating a leakage index over a period of 5 min at sites exhibiting higher fluorescence by using the formula,

(1) $$\begin{array}{l} \text{Leakage index}=\frac{\;Intensity\;\left(tn\right)-\;Intensity\;\left(t0\right)}{Intensity\;\left(t0\right)}, \end{array}$$

where *n* = 1, 2, 3, 4, 5 min and t_0_ is 5–10 s after the administration of TRITC-Dextran.

The leakage index was calculated for 6 regions of interest (ROI) identified near 3 arteries and 3 veins in each animal studied. At each time point the leakage index was normalized to the baseline value for any given animal. For all animals the selected ROIs were of comparable size and free from crowding by any smaller and underlying vessels. The boundaries of ROIs were drawn on the baseline image and placed at the same locus on the images captured at later time points for each animal. Image analysis and processing was performed using ImageJ (National Institutes of Health, Bethesda, MD) (29). Intergroup significance in mean differences in the leakage indices was determined by two-way ANOVA followed by Dunnett's multiple comparisons test with adjusted *p* ≤ 0.0007 considered to be significant. For each group, the rate of leakage was determined from leakage indices over 5 min by using a linear regression model and mean best-fit values ± standard error. Mann-Whitney unpaired *t*-test was used to compare the rate of leakage between groups with *p* ≤ 0.05 considered to be significant.

## Results

**Figure 2:**
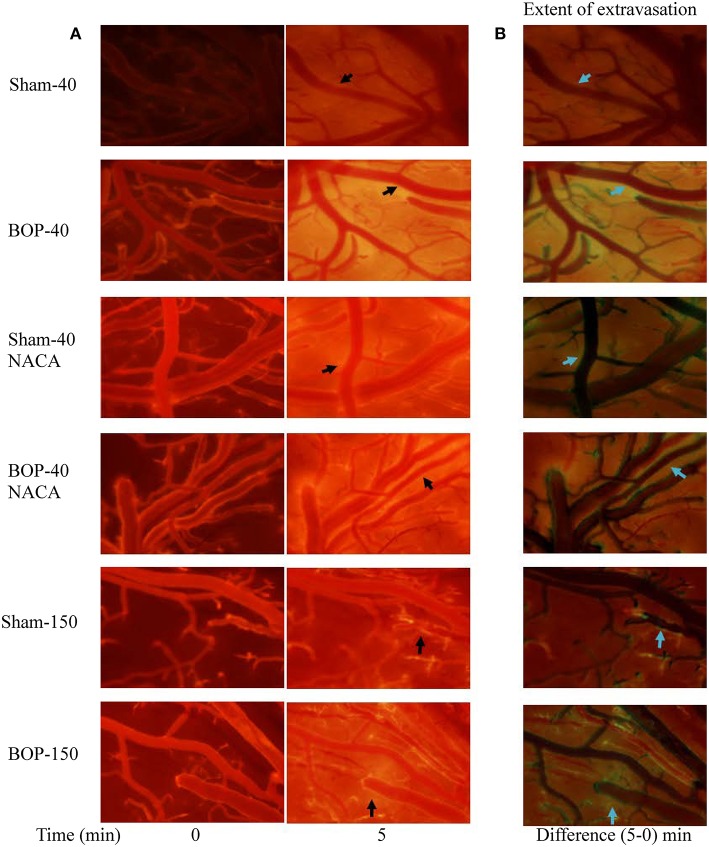
Representative images of pial microvessels after intravenous administration of a fluorescent probe (TRITC-Dextran). **(A)** Images acquired immediately and 5 min after the injection. **(B)** Difference of images captured at 5 min and immediately after the injection- indicative of extravasation of the fluorescent probe through BBB. Arrows shows examples of areas where extravasation of the probe was observed.

**Figure 3:**
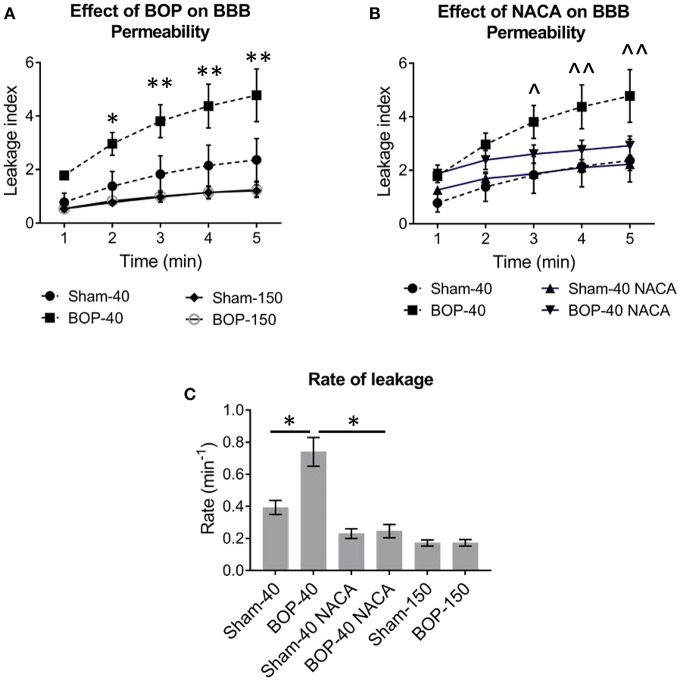
The effect of blast and NACA administration on the permeability of blood-brain barrier (BBB) in pial vessels. **(A)** Leakage of 40 kD TRITC-Dextran and 150 kD TRITC-Dextran. **(B)** NACA administration attenuates the leakage of 40 kD TRITC-Dextran in blast group. **(C)** The rate of leakage of fluorescent probes through pial vessels with or without exposure to blast and the effect of NACA on the rate of leakage of 40 kD probe. Intergroup significance in mean differences was determined by two-way ANOVA followed by Dunnett's multiple comparison test. *, **; and ∧, ∧∧ represent significant differences between Sham-40 and BOP-40, and BOP-40 and BOP-40 NACA, respectively, with adjusted p-values of * = 0.0004, ∧∧ = 0.0007, and **, ∧ = 0.0001. The rate of leakage was determined by using a linear regression model and best-fit values ± standard error. The R square values for the goodness of fit were ≥ 0.922 and there was no significant deviation from linearity. Mann-Whitney unpaired t-test was used to compare the rate of leakage between groups (Sham-40 vs. BOP-40 and BOP-40 vs. BOP-40 NACA) with p ≤ 0.05 considered to be significant.

Exposure to repetitive blasts led to a compromise in the integrity of BBB which was seen in an acute phase (2–3 h) after the injury. Qualitatively, the differences in the intensities of images captured at 5 min and baseline presented the extent of leakage through the BBB as shown by representative images from each group in Figure 2. The vessels in BOP-40 group appeared to be more permeable to the fluorescent probe than any other groups as shown in the “difference image” in Figure 2B. The leakage in the Sham-150 and BOP-150 groups was substantially lower than groups that received 40 kD TRITC-Dextran. The brightness of “difference image” in both NACA treated groups was diminished in comparison to the Sham-40 and BOP-40 groups. Figure 3 shows leakage index and the rate of leakage estimated over 5 min after the injection of TRITC-Dextran. The index values were significantly higher in BOP-40 group in comparison to all other groups. NACA treatment reduced leakage through BBB in both sham and blast exposed animals. There was no significant difference in the permeability of 150 kD probe between the sham and blast-exposed groups and the index values were significantly smaller than other groups.

## Discussion

Repeated exposures to blast alter BBB integrity in rats resulting in size specific increase in permeability. In our study, three exposures to 110 kPa BOP contributed to a significant extravasation of 40 kD probe and an unremarkable leakage of 150 kD probe from pial vessels. Therefore, the efficacy of NACA in conferring protection against BBB breakdown and ameliorating the leakage of 40 kD probe through the barrier was assessed.

Primary injury caused by bTBI is followed by a cascade of processes resulting in secondary damage. The events that follow the primary injury include an imbalance in the demand to supply ratio of glucose, lipid peroxidation, inefficient free radical scavenging, and inflammation, which inflict further damage to the already injured brain (30, 31). A potential direct consequence of the primary insult from mechanical forces and the spike in ICP caused by BOP is the rupturing of microvessels in the brain (32, 33). Petechial hemorrhaging can induce an increase in intracellular Ca^2+^, which in turn triggers neuroinflammatory cascades and increases the accumulation of reactive oxygen species (ROS) (34, 35). ROS accumulation triggers oxidative stress, alteration of TJ, and activation of matrix metalloproteinases which mediate increase in BBB permeability (7, 36). An intervention with an antioxidant at an opportune time can circumvent the progression of inflammatory cascades and prevent further damage to the brain. Therapeutically-directed restoration of BBB permeability after injury may be more effective if the intervention is provided when BBB is more permeable for the drugs to cross the barrier.

TBI has been shown to increase oxidative stress and deplete endogenous antioxidants like GSH (37–41). GSH has a significant role in maintenance and proper function of BBB integrity (42). Treatment with GSH depleting agents resulted in an increase in BBB permeability and replenished GSH levels were associated with normalization of the permeability (42). Both NAC and NACA play a role in scavenging of free radicals and enhancing the synthesis of GSH, with NACA being more efficient due to its enhanced bioavailability (24). NACA significantly increased GSH and diminished levels of malondialdehyde (byproduct of lipid peroxidation) in immortalized endothelial cell line from rat brain capillaries in a model of oxidative stress induced BBB disruption (43). In a rat TBI model, NACA was shown to reduce inflammation following blast injury (24, 44) and increase working memory performance compared to controls after penetrating brain injury (45). While post-blast NACA treatment in rats attenuated the blast-induced elevation of ICP, the effect of NACA was more robust when administered prior to the delivery of injury (16). NACA was effective in improving and preserving mitochondrial bioenergetics after TBI and spinal trauma (45). In a focal penetrating brain injury model, NACA treatment reduced neuronal degeneration and apoptosis and simultaneously increased the levels of the mitochondrial antioxidant manganese superoxide dismutase (2). Even though NACA has shown promising outcomes in preclinical studies, it would be further advantageous to use pharmacological approaches that target more than one mechanism of oxidative neurodegeneration (46). A combination of mechanistically complimentary antioxidants can potentially interrupt the progression of the cascade of processes leading to oxidative neurodegeneration.

The study presented here demonstrates that BBB integrity is compromised in the acute phase after BOP exposure and that NACA treatment at a single dose may significantly protect against BBB breakdown. A longitudinal study mapping BBB damage over time and the natural recovery (if any) would be of interest in characterizing the effect of bTBI on BBB. Additionally, exposure to different blast regimens would aid in developing a model of BBB compromise at various blast intensities and repetitions. Although such extensions of the investigation were not within the scope of the study, characterization of BBB damage will be vital in determining the optimal therapeutic window for intervention with NACA/combination of antioxidants to potentially improve the outcome.

## Data Availability

All datasets generated for this study are included in the manuscript and/or the supplementary files.

## Ethics Statement

The study protocol was reviewed and approved by the Walter Reed Army Institute of Research/Naval Medical Research Center Institutional Animal Care and Use Committee in compliance with all applicable Federal regulations governing the protection of animals in research. The experiments reported herein were conducted in compliance with the Animal Welfare Act and per the principles set forth in the Guide for Care and Use of Laboratory Animals, Institute of Laboratory Animals Resources, National Research Council, National Academy Press, 2011.

## Author Contributions

UK contributed toward study design, manuscript preparation and revision, image, data and statistical analysis. RA contributed toward study design, image analysis and manuscript revision. SZ contributed toward manuscript preparation and image analysis. AQ performed image analysis. SA contributed toward data analysis and presentation, manuscript revision and is a content expert. RM contributed toward study design. MC contributed toward study design.

### Conflict of Interest Statement

The authors declare that the research was conducted in the absence of any commercial or financial relationships that could be construed as a potential conflict of interest.
